# A Systematic Review and Meta-Analysis of Laparoscopic Ligation of the Inferior Mesenteric Artery for the Treatment of Type II Endoleaks

**DOI:** 10.31083/j.rcm2306208

**Published:** 2022-06-01

**Authors:** Vangelis Bontinis, Andreas Koutsoumpelis, Alkis Bontinis, Argirios Giannopoulos, Kiriakos Ktenidis

**Affiliations:** ^1^Department of Vascular Surgery, Aristotle University of Thessaloniki, AHEPA University General Hospital, 54621 Thessaloniki, Greece

**Keywords:** type II endoleak, laparoscopic ligation, IMA ligation, inferior mesenteric artery

## Abstract

**Objectives:**

Type II endoleak (T2E), often generated by persistent retrograde flow through the inferior mesenteric artery (IMA) is the most frequent complication following endovascular aortic aneurysm repair (EVAR). T2E treatment revolves around transarterial and translumbar embolization of the feeding artery and/or sac, with mediocre results. The aim of this study is to assess the safety feasibility and efficacy of laparoscopic IMA ligation for the treatment of T2E.

**Methods:**

We conducted a systematic electronic research on Medline, Scopus, EMBASE, and Cochrane Library according to Preferred Reporting Items for Systematic Review and Meta-Analysis protocol (PRISMA) for articles published up to February 2022, describing laparoscopic IMA ligation for the treatment of T2E. Publications describing hand assisted or prophylactic IMA ligation were excluded. A metanalysis was performed utilizing both the random and common effects model and the DerSimonian and Laird method. Additionally, we carried out a post hoc power analysis.

**Results:**

Fifteen studies, including one prospective case series (CS), five retrospective CS and nine case reports, including 33 patients (91% male) met the inclusion criteria. The mean abdominal aortic aneurysm diameter at the time of diagnosis was 58.8 mm. The mean operational duration was 117.5 minutes. The mean follow-up for the included studies was 17 months. The mean reported time of T2E identification was 9.1 months post-intervention, while the mean reported aneurysmal sac diameter increase at the time of diagnosis was 11.5 mm. T2E type a (T2aE) and type b (T2bE) patterns were 57.6% and 42.4% respectively. Six CS incorporating 24 patients were included in the meta-analysis. The pooled technical success and postoperative mortality rates are 100% (95% CI: 93.13–100), ($I^{2}$ = 0.0%, *p* = 0.99) (power = 99%) and 0.00% (95% CI: 0.00–6.87) ($I^{2}$ = 0.0%, *p* = 0.99). The pooled reintervention and conversion to open surgical repair rates are 15.08% (95% CI: 0.79–37.28), ($I^{2}$ = 0.0%, *p* = 0.66) (power = 13.6%), and 0.69% (95% CI: 0.00–14.80) ($I^{2}$ = 0.0%, *p* = 0.99) (power = 7.05%) respectively.

**Conclusions:**

We demonstrated the safety and feasibility of IMA ligation for the treatment of T2E. Definitive conclusions about its efficacy cannot be drawn due to underpowered results warrantying further research. Identification and proper classification of T2E remain an obstacle affecting treatment outcomes and reintervention rates throughout the entire spectrum of available treatments.

## 1. Introduction

Endoleaks refer to ineffective sealing of the aneurysmal sac following endovascular aneurysm repair (EVAR), manifesting as persistent blood flow into the aneurysm. Five types of endoleaks have been described ranging from type I to V [1]. Type II (T2E) endoleaks are defined as persistent perfusion of the aneurysmal sac through collateral arterial circulation. The arteries involved include the inferior mesenteric artery, lumbar arteries, and sacral artery. T2E is further classified as Type IIa (to-and-fro flow type) where there is a single vessel involved and Type IIb where more than one vessels are involved [2].

T2E endoleak is the most common type of endoleak detected in up to 25% of post-EVAR computed tomography angiographies (CTA) [3]. T2E follows a benign course with a reported annual spontaneous resolution rate of up to 80%. Despite that, T2E is responsible for about 40% of post-EVAR re-intervention [4].

Given the low aneurysm rupture risk of <1%, the indications for T2E treatment include symptomatic sac expansion and progressive sac enlargement of ≥5 mm [5]. Treatment options include transarterial, translumbar and transcaval embolization of the feeding arteries and/or the aneurysmal sac as well as open surgical or laparoscopic interventions [6].

The most frequently employed techniques are transarterial and translumbar embolization. Although both techniques are technically feasible with high rates of technical success of about 85%, clinical failure is common with continuous aneurysmal sac expansion in up to 60% of the treated patients while a recent meta-analysis by Guo *et al*. [7] demonstrated no statistically significant differences between the two interventions [8]. Finally, the indication for open surgical repair (conversion) is failed endovascular therapy, a practice carrying non-negligible complication and mortality rates [9].

Due to the lack of a validated algorithm, laparoscopic surgery sits in the gray area between endovascular repair and open surgical repair. The aim of this study is to assess the safety, feasibility, and efficacy of laparoscopic inferior mesenteric artery ligation in the treatment of T2E.

## 2. Methods

### 2.1 Information Sources and Search Strategy 

A systematic review was conducted according to the recommendations of the Preferred Reporting Items for Systematic Reviews and Meta-Analyses (PRISMA) statement [10]. The systematic review protocol was not registered while it can be accessed at a reasonable time frame by contacting the corresponding author. A systematic electronic search on Medline, Scopus, EMBASE, and Cochrane Library was conducted by two independent researchers A.B and V.B for articles published up to February 2022. Controlled vocabulary supplemented with keywords was used to search for studies describing laparoscopic IMA ligation in the treatment of T2E. The terms and term combination for conducting this research included: “type 2 endoleak”, “inferior mesenteric artery”, “type 2 endoleak IMA”, “type 2 endoleak laparoscopic”, “type 2 endoleak ligation” (**Supplementary Figs. 1–3**).

### 2.2 Selection Process and Data Collection Process 

The method of data collection involved two independent researchers V.B and A.K reviewing the titles and abstracts of the retrieved literature. Publications whose titles and abstracts met the inclusion criteria were obtained in full, analyzed, and processed using the same terms by both researchers. The rest were excluded. When disagreement arose, a consensus was reached through discussion. There were no language, gender, age, or clinical status limitations.

### 2.3 Eligibility Criteria

Inclusion criteria

(1) Publications describing laparoscopic ligation of IMA in the treatment of T2E.

(2) Retrospective and prospective case series, case reports, randomized control trials, registry reports, and reports in the form of letters to the editor were all included.

Exclusion criteria

(1) Publications describing hand assisted laparoscopic ligation of IMA for the treatment of T2E were excluded.

(2) Publications describing prophylactic ligation of IMA during a laparoscopic or robotic aneurysm repair were excluded.

(3) Publications not reporting information on a minimum of one primary outcome were excluded.

### 2.4 Data Items 

Primary endpoints include, technical success, reintervention rate and conversion to open surgical repair. Secondary endpoints include postoperative mortality and mean operative time.

### 2.5 Definitions

Technical failure was defined as the inability to identify and ligate the IMA. Reintervention was defined as re-operation due to persistent T2E. IMA related reintervention was defined as a reintervention associated with the successful ligation of IMA per se. Postoperative mortality was defined as death of any etiology occurring within 30 days after the intervention.

### 2.6 Effect Measures and Synthesis Methods

Descriptive statistics were reported for all the included studies in the systematic review and presented as proportions %. In the metanalytic process six cases series including twenty-four patients were included [11, 12, 13, 14, 15, 16]. A meta-analysis of aggregate data for technical success, reintervention, and conversion rates were implemented. Due to the small sample sizes and rare occurrence of events we utilized the inverse variance method of transformed proportions and the Freeman Tukey double arcsine transformation for deriving pooled estimates.

We performed both random-effects and common effects model estimation using DerSimonian and Laird Random method, results were presented accordingly. A formal statistical test for heterogeneity using the $I^{2}$ test was undertaken. Heterogeneity was defined as low (0%–25%), moderate (25%–50%) and high (>50%).

Prediction intervals (PI) were calculated for all endpoints and presented accordingly. All metanalyses are displayed visually with the use of forestplots.

Due to the small number of patients included in the meta-analysis a post hoc power analysis was undertaken for the three primary endpoints. The results are presented as proportions and visualized with the use of a fireplot.

For the statistical analysis we used RStudio (R Foundation for Statistical Computing, Vienna, Austria, v 4.1.3).

## 3. Results

### 3.1 Baseline Study Characteristics 

During the systematic review, fifteen studies including 33 individuals (30 male) met the inclusion criteria. One prospective case series, five retrospective case series and nine case reports were included [11, 12, 13, 14, 15, 16, 17, 18, 19, 20, 21, 22, 23, 24, 25]. Twelve studies reported on laparoscopic ligation of IMA, while three studies on robotic interventions [12, 14, 20]. The mean abdominal aortic aneurysm diameter at the time of diagnosis was reported by fourteen studies and it was 58.8 mm [11, 12, 13, 14, 15, 16, 17, 18, 19, 20, 21, 23, 24, 25] (**Supplementary Fig. 4**).

The mean operational duration was reported by thirteen studies, and it was 117.5 minutes [11, 12, 13, 14, 15, 16, 17, 18, 19, 20, 23, 24, 25]. The mean follow-up for the included studies was 17 months. The mean time of T2E identification was reported by eleven studies and it was 9.1 months post-intervention [12, 16, 17, 18, 19, 20, 21, 22, 23, 24, 25]. Out of the 33 T2E included, 32 were identified during follow-up examination, while in one case symptoms lead to the identification of the endoleak [11]. The mean reported aneurysmal sac diameter increase at the time of diagnosis was 11.5 mm. T2E type a (T2aE) and type b (T2bE) patterns were reported in 57.6% and 42.4% respectively. Failed endovascular interventions preceding laparoscopic or robotic ligation were reported by fourteen studies producing a 43.5% (10/23) rate of previously failed interventions [11, 12, 14, 15, 16, 17, 18, 19, 20, 21, 22, 23, 24, 25]. Fourteen out of fifteen studies reported on previous endovascular interventions for the treatment of T2E with a reported trans-arterial or translumbar embolization failure rate of 43.5% (10/23) [11, 12, 14, 15, 16, 17, 18, 19, 20, 21, 22, 23, 24, 25]. Whether combined procedures were undertaken during IMA ligation were reported by 14 studies [11, 12, 14, 15, 16, 17, 18, 19, 20, 21, 22, 23, 24, 25].

Seven studies including twelve patients reported on undertaking combined procedures [14, 15, 17, 18, 19, 23, 25]. In about 75% (9/12) of cases, concomitant ligation of lumbar or internal iliac arteries occurred. Three studies reported on primary direct puncture aneurysmal sac embolization, while one study reported on secondary sac embolization during reintervention [14, 17, 18, 23] (Table 1 (Ref. [11, 12, 13, 14, 15, 16, 17, 18, 20, 21, 22, 23, 24, 25]), **Supplementary Table 1**).

**Table 1.** **Baseline study characteristics**.

Study	Patients (n)	Type	Operative time (minutes)	T2E (type)	AAA diameter (mm)	Follow up (months)	Imaging diagnostics
Porta 2020 [11]	3	PCS	58	IIa	59	15	CTA
Norberto 2019 [17]	1	CR	132	IIa	62	12	CTA
Morelli 2019 [12]	2	RCS	183	IIb	62.5	N/A	CTA
Fadda 2017 [18]	1	CR	N/A	IIb	57	0.2	CTA
Piffaretti 2017 [13]	10	RCS	97	8IIa,2IIb	60	46	CTA
							CEUS
Zou 2014 [22]	1	CR	50	IIb	70	0.23	CTA
Lin 2012 [14]	2	RCS	221	IIb	55	38.5	N/A
Linsen 2011 [15]	5	RCS	140.4	4IIb,1IIa	61.4	50	N/A
Lin 2009 [20]	1	CR	249	IIa	61	3	CTA
Feezor 2006 [21]	1	CR	n/a	IIa	67	17	CTA
Zhou 2006 [22]	1	CR	n/a	IIa	NA	N/A	CTA
Karkos 2005 [23]	1	CR	80	IIb	9.3	0.23	CTA
Ho 2004 [24]	1	CR	60	IIa	60	N/A	CTA
Richardson 2003 [16]	2	RCS	85	IIa	55	22	CTA
Wisselink 2000 [25]	1	CR	130	IIb	65	0.13	CTA

*Abbreviations: RCS, retrospective case series; PCS, prospective case series; CR, case report; T2E, type II endoleak; CTA, computed tomography angiography; CEUS, contrast-enhanced ultrasound; AAA, abdominal aortic aneurysm.

### 3.2 Technical Success

**Fig. 1.**
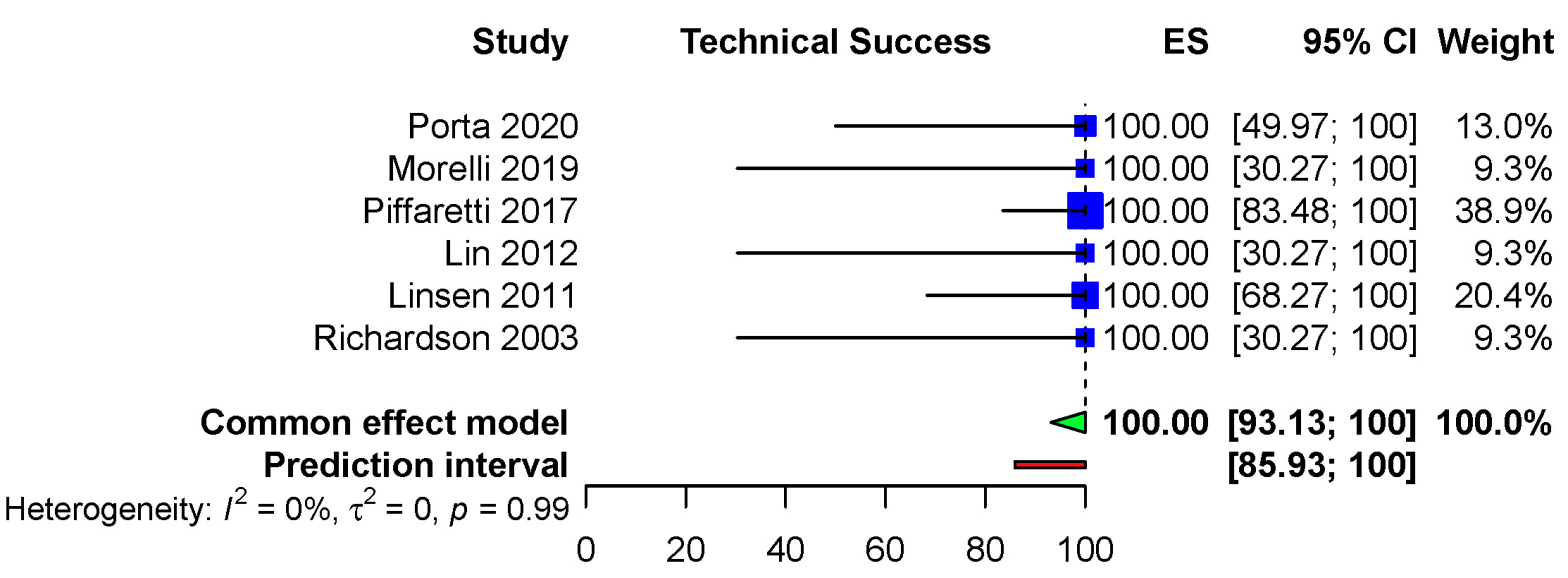
**Fixed effects model—Technical success**.

The crude technical success rate was reported by all included studies. The reported technical success rate is 100% (33/33). The pooled technical success rate for the six included studies in the meta-analysis is 100% (95% CI: 93.13–100) ($I^{2}$ = 0.0%, *p* = 0.99), PI (85.93–100) [11, 12, 13, 14, 15, 16] (Fig. 1).

### 3.3 Reintervention Rate

**Fig. 2.**
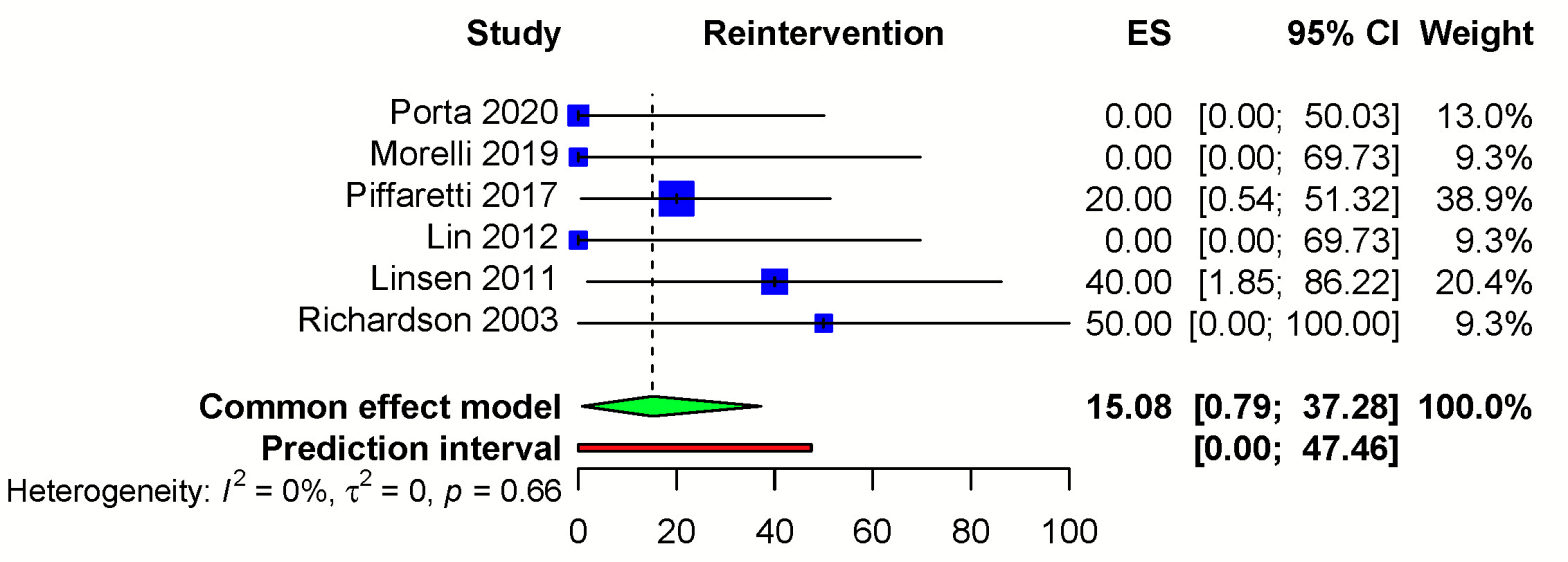
**Fixed effects model—Reintervention rate**.

The crude reintervention rate for the fifteen included studies is 18.2% (6/33). The pooled reintervention rate for the six included studies is 15.08% (95% CI: 0.79–37.28) ($I^{2}$ = 0.0%, *p* = 0.66), PI (0.00–47.46) (Fig. 2).

The crude and pooled IMA related reintervention rates are 3% (1/33) and 0% (95% CI: 0.00–10.73) ($I^{2}$ = 0.0%, *p* = 0.65), PI (0.00–18.75).

### 3.4 Reintervention Rates According to T2E Type and Primary Embolization

T2aE group included seven studies reporting exclusively on patients treated for T2aE [11, 14, 16, 17, 21, 22, 24]. Additionally, eight patients extracted from the study by Piffaretti *et al*. [13], and a single patient extracted by the study of Linsen *et al*. [15], all treated for T2aE were added to this subgroup.

T2bE group included five studies exclusive reporting on T2bE [12, 14, 18, 19, 23]. Four patients by the study of Linsen *et al*. [15] and two patients by Piffaretti *et al*. [13] were also added to this subgroup.

The crude T2aE reintervention rate is 5.26% (1/19), while the T2bE reintervention rate is 35.7% (5/14). Three studies where primary embolization was undertaken reported a reintervention rate of 0% (0/4).

### 3.5 Postoperative Mortality 

No reported incidents of post-operative mortality were recorded. The crude postoperative mortality rate is 0% (0/33) while the pooled postoperative mortality is 0.00% (95% CI: 0.00–6.87) ($I^{2}$ = 0.0%, *p* = 0.99), PI (0.00–14.07).

### 3.6 Conversion to Open Surgical Repair 

**Fig. 3.**
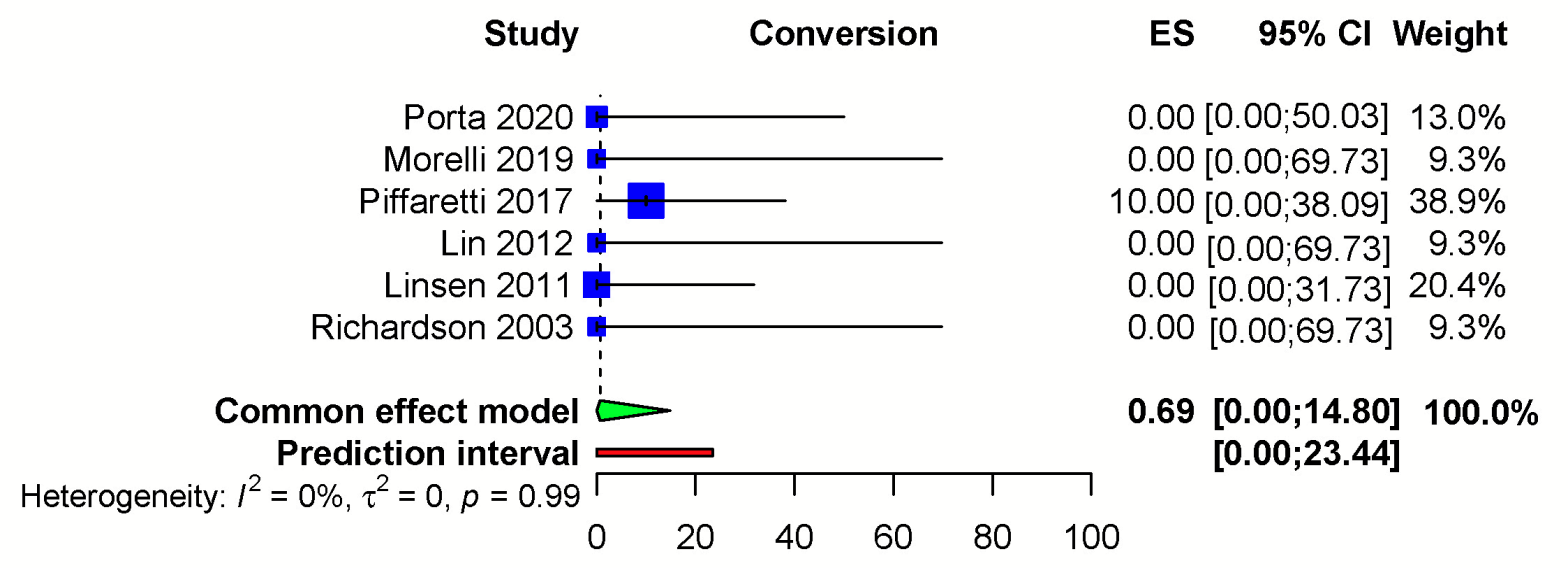
**Fixed effects model—Conversion to open surgical repair**.

One incident of open surgical conversion was reported due to persistent T2bE leading to a T3E producing a crude conversion rate of 3% (1/33). The pooled conversion to open surgical repair rate is 0.69% (95% CI: 0.00–14.80) ($I^{2}$ = 0.0%, *p* = 0.99), PI (0.00–23.44) (Fig. 3).

### 3.7 Mean Operative Time 

Twelve studies including 30 patients reported on mean operative time [11, 12, 13, 14, 15, 16, 17, 20, 22, 23, 24, 25]. The mean operative time for the included studies is 117.5 minutes. Seven studies including ten patients reported on isolated IMA clipping with a mean operative time of 77 minutes [11, 15, 16, 17, 19, 23, 24]. Four studies including ten patients reported on additional lumbar artery ligation with a mean operative time of 178.5 minutes [12, 14, 20, 25]. The mean operative time for three studies where primary aneurysmal sac embolization was undertaken is 108 minutes.

### 3.8 Power Analysis

**Fig. 4.**
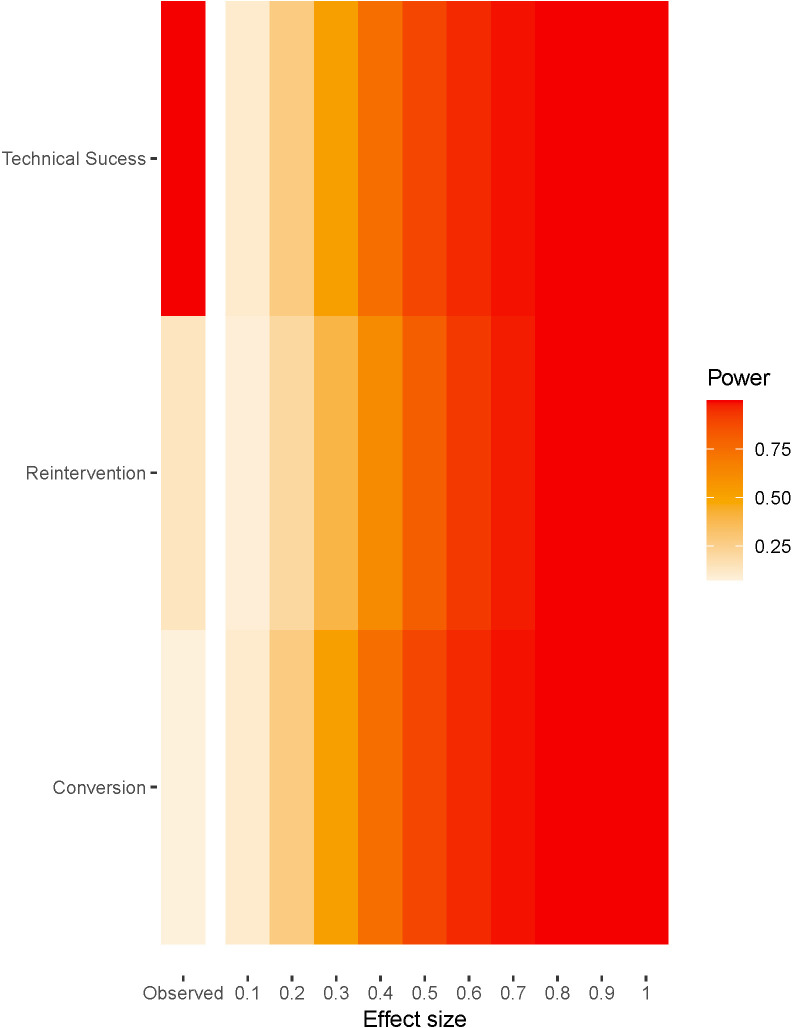
**Fireplot—Statistical power of the meta-analyses**.

We performed a post hoc power analysis for the three primary endpoints. The statistical power for the technical success, conversion, and reintervention metanalyses are 99%, 7.5% and 13.6% respectively. Conversion and reintervention endpoints are severely underpowered a fact also underlined by their wide confidence intervals (CI) and prediction intervals (PI) (Fig. 4).

## 4. Discussion 

The pooled technical success and reintervention rates are 100% and 15.08% respectively. The pooled postoperative mortality and conversion to open surgical repair rates are 0% and 0.69% respectively. The mean operative time for the fourteen included studies is 117.5 minutes.

Type II endoleak is the most common type of endoleak with a reported six-month post-EVAR prevalence of up to 15% while it is responsible for most post-EVAR reinterventions. The presence of a patent IMA and the number of patent lumbar arteries are well known predictors for the development of T2E. Guo* et al*. [3] in their metanalysis of 36.588 participants demonstrated almost two times higher odds for developing T2E in the presence of a patent IMA and three times higher odds regarding the number of patent lumbar arteries [26].

The benign course of T2E is well documented, with most T2E spontaneously resolving and a low rupture risk for those failing to resolve. Major indications for treatment include aneurysmal expansion and/or symptoms attributed to aneurysmal growth (grade 2C evidence level) [6].

According to the published guidelines and despite the absence of a validated treatment algorithm, the initial T2E treatment involves transarterial, translumbar or transcaval embolization. Should initial treatment fail, laparoscopic ligation or open conversion follows (grade 2C evidence level).

Transarterial embolization technical success rates range from 60% to 80% with reported reintervention rates of about 40% [27]. The modest technical success rates showcased by transarterial embolization are mainly attributed to the often-torturous nature of the collateral circulation and its demanding catheterization. On the other hand, despite the significantly improved technical success rates showed by translumbar embolization, reintervention rates are analogous to that of its transarterial counterpart. Guo *et al*. [7] in their metanalysis of translumbar versus transarterial embolization, although demonstrating thirteen times higher odds regarding technical failure for transarterial embolization, produced no statistically significant differences between the two techniques regarding clinical success.

In our review, we showed excellent technical success and acceptable re-intervention rates. Most reinterventions were due to T2bE endoleaks or T2bE endoleaks wrongly diagnosed as T2aE. In particular, the study by Piffaretti *et al*. [13] reported two instances of persistent endoleaks. The first case was a T2bE where not all involved lumbar arteries were identified and ligated, leading to a type 3 endoleak (T3E) and an open conversion. In the second case, a T2bE was misclassified as T2aE leading to a successful trans-arterial embolization (TAE) [13]. Two instances of persistent endoleaks leading to reinterventions were also reported by Linsen *et al*. [15]. In one case, laparoscopic ligation of spared lumbar arteries was undertaken, while in the second instance coil embolization was required [15]. Richardson *et al*. [16] reported a reintervention due to sparing of a feeding IMA branch requiring ligation without providing information on whether this came as a result of an IMA anatomical variation or unsuccessful operational planning. Finally, Wisselink *et al*. [25] reported a case of a persistent T2bE leading to reintervention and laparoscopic lumbar artery ligation.

The gold standard around post-EVAR surveillance is computed tomography angiography (CTA). The reported sensitivity and specificity of CTA in endoleak detection is about 83% and 100% respectively [28]. Methods such as magnetic resonance angiography (MRA), contrast-enhanced ultrasound (CEUS) and 3-D CEUS (CEtUS) are reported to be equally efficient if not superior compared to CTA.

Despite the high sensitivity in detecting the presence of endoleaks portrayed by the entirety of imaging methods at our disposal, the distinction between different types of endoleaks (which often coexist) is somewhat demanding. This is particularly true for T2E because of their low flow nature and the involvement of multiple collateral arteries (T2bE).

The inferiority of CTA compared to its alternatives in detecting and classifying T2E is advocated by numerous authors. In several reported studies, MRA was able to identify 10% to 30% more T2E compared to CTA while similar results are reported for the CEUS and CEtUS imaging modalities [2, 29, 30]. In our review, a single study reported endoleak detection by CEUS while the remaining fourteen studies followed a CTA diagnostic regimen [13].

In our review, 83.3% (5/6) of the reinterventions undertaken involved T2bE and resulted from either preoperative unsuccessful mapping of inflow and/or outflow collaterals, or because of T2E misclassification (T2bE wrongfully diagnosed as T2aE). The genuine reintervention rate involving IMA ligation is 0% since.

According to the literature, transarterial embolization requires prolonged fluoroscopy compared to both translumbar and transcaval techniques. In their retrospective case series, Yang *et al*. [31] demonstrated almost a fourfold prolongation of fluoroscopy time for transarterial embolization compared to direct sac puncture embolization (11 minutes versus 42 minutes). In our study the overall mean reported operative time is 117.5 minutes. When the operation involved T2aE (isolated IMA engagement), operative duration reached 77 minutes while regarding T2bE we observed a 131.1% increase in procedural duration (178.5 minutes).

Open surgical alternatives to endovascular and minimally invasive techniques include either open repair with graft explanation or graft preservation. Open surgical repair with graft explanation carries high reported post-operative mortality rates of about 10% and 31% respectively [32]. Although graft preservation techniques display improved mortality and morbidity rates of 4% and 14%, their results are barely comparable to those reported for both the minimally invasive and endovascular techniques.

## 5. Limitations

The main limitations of this review are the limited number of patients included and the retrospective nature of the studies, affecting both the robustness of our results and leaving the study vulnerable to sampling error and selection bias.

## 6. Conclusions

This review and meta-analysis demonstrated the safety and feasibility of IMA ligation for the treatment of T2E. Definitive conclusions about its efficacy cannot be drawn due to underpowered results warrantying further research. Identification and proper classification of T2E remain an obstacle affecting treatment results and reintervention rates through the entire spectrum of available treatments.

## Supplementary Material

Supplementary material associated with this article can be found, in the online version, at https://doi.org/10.31083/j.rcm2306208.
