# Supplementary material for: A Systematic Review and Meta-Analysis of Laparoscopic Ligation of the Inferior Mesenteric Artery for the Treatment of Type II Endoleaks
Source: Rev Cardiovasc Med. 2022 Jun 1;23(6):208. doi: 10.31083/j.rcm2306208 (PMC11273796; doi:10.31083/j.rcm2306208)
Supplement: Supplementary file 1 [file 2153-8174-23-6-208-s1.zip › Supplementary Table 1 – Primary and Secondary endpoint results .docx]

| Study | Technical Success | | Conversion | | Reintervention | | Thirty-day Mortality | |
| --- | --- | --- | --- | --- | --- | --- | --- | --- |
|  | Event | Total | Event | Total | Event | Total | Event | Total |
| Porta 2020 [11] | 3 | 3 | 0 | 3 | 0 | 3 | 0 | 3 |
| Norberto 2019 [17] | 1 | 1 | 0 | 1 | 0 | 1 | 0 | 1 |
| Morelli 2019 [12] | 2 | 2 | 0 | 2 | 0 | 2 | 0 | 2 |
| Fadda 2017 [18] | 1 | 1 | 0 | 1 | 0 | 1 | 0 | 1 |
| Piffaretti 2017 [13] | 10 | 10 | 1 | 10 | 2 | 10 | 0 | 10 |
| Zou 2014 [22] | 1 | 1 | 0 | 1 | 0 | 1 | 0 | 1 |
| Lin 2012 [14] | 2 | 2 | 0 | 2 | 0 | 2 | 0 | 2 |
| Linsen 2011 [15] | 5 | 5 | 0 | 5 | 2 | 5 | 0 | 5 |
| Lin 2009 [20] | 1 | 1 | 0 | 1 | 0 | 1 | 0 | 1 |
| Feezor 2006 [21] | 1 | 1 | 0 | 1 | 0 | 1 | 0 | 1 |
| Zhou 2006 [22] | 1 | 1 | 0 | 1 | 0 | 1 | 0 | 1 |
| Karkos 2005 [23] | 1 | 1 | 0 | 1 | 0 | 1 | 0 | 1 |
| Ho 2004 [24] | 1 | 1 | 0 | 1 | 0 | 1 | 0 | 1 |
| Richardson 2003 [16] | 2 | 2 | 0 | 2 | 1 | 2 | 0 | 2 |
| Wisselink 2000 [25] | 1 | 1 | 0 | 1 | 1 | 1 | 0 | 1 |

Supplementary Table 1. Primary and Secondary endpoint results.
