# Supplementary figures and images for: A Systematic Review and Meta-Analysis of Laparoscopic Ligation of the Inferior Mesenteric Artery for the Treatment of Type II Endoleaks
Source: Rev Cardiovasc Med. 2022 Jun 1;23(6):208. doi: 10.31083/j.rcm2306208 (PMC11273796; doi:10.31083/j.rcm2306208)

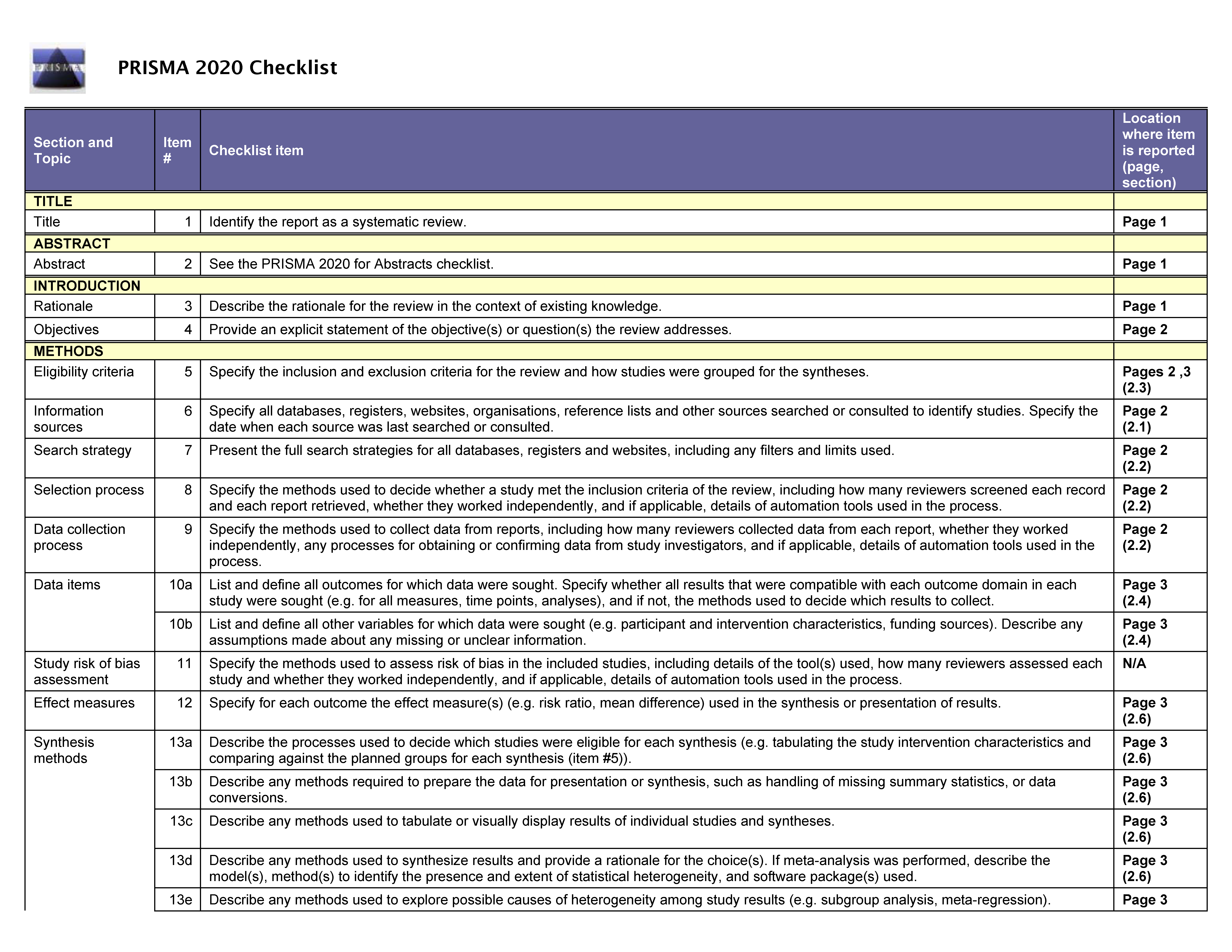

Supplement: Supplementary file 1 [file 2153-8174-23-6-208-s1.zip › Supplementary Figure 1 - PRISMA CHECKLIST.tiff]

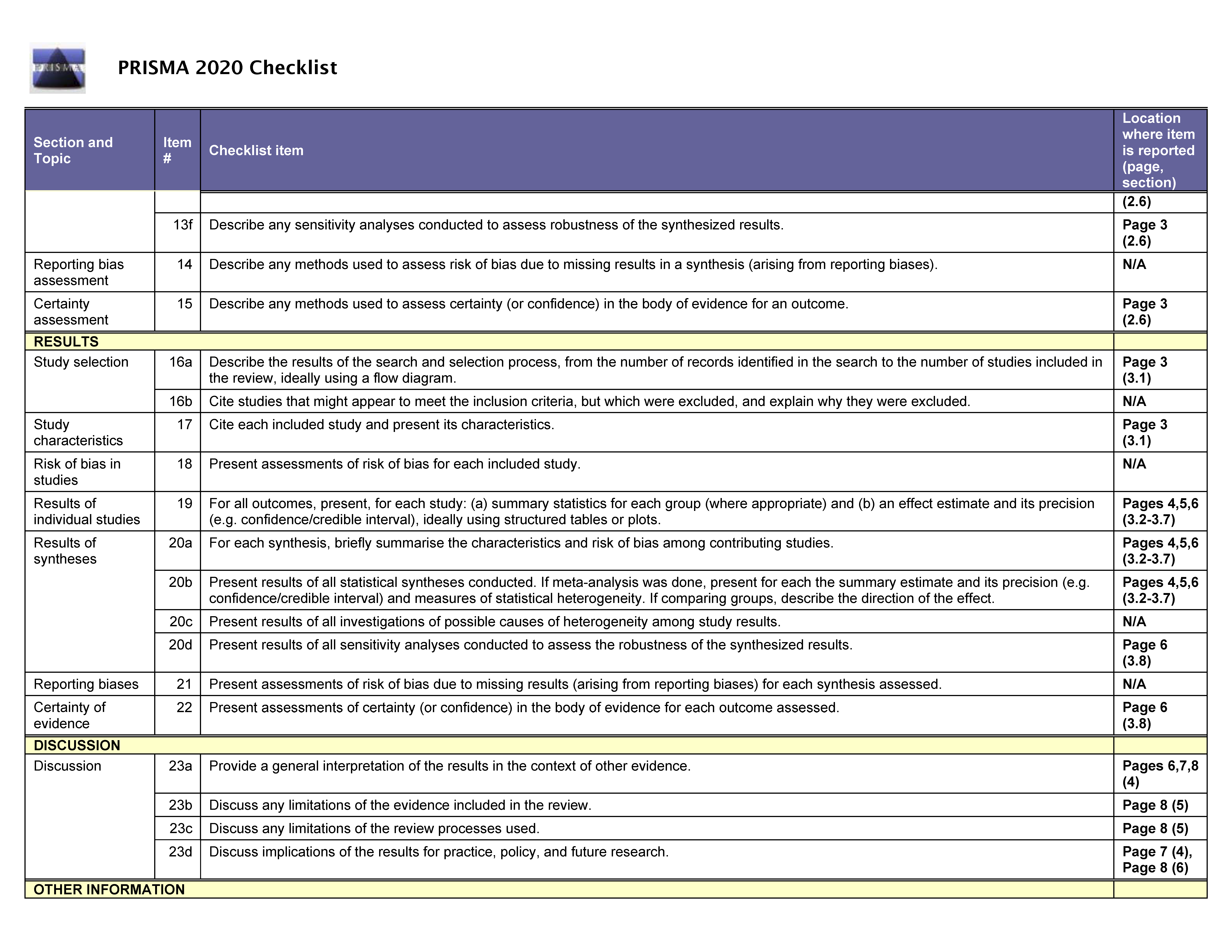

Supplement: Supplementary file 1 [file 2153-8174-23-6-208-s1.zip › Supplementary Figure 2 - PRISMA CHECLIST.tiff]

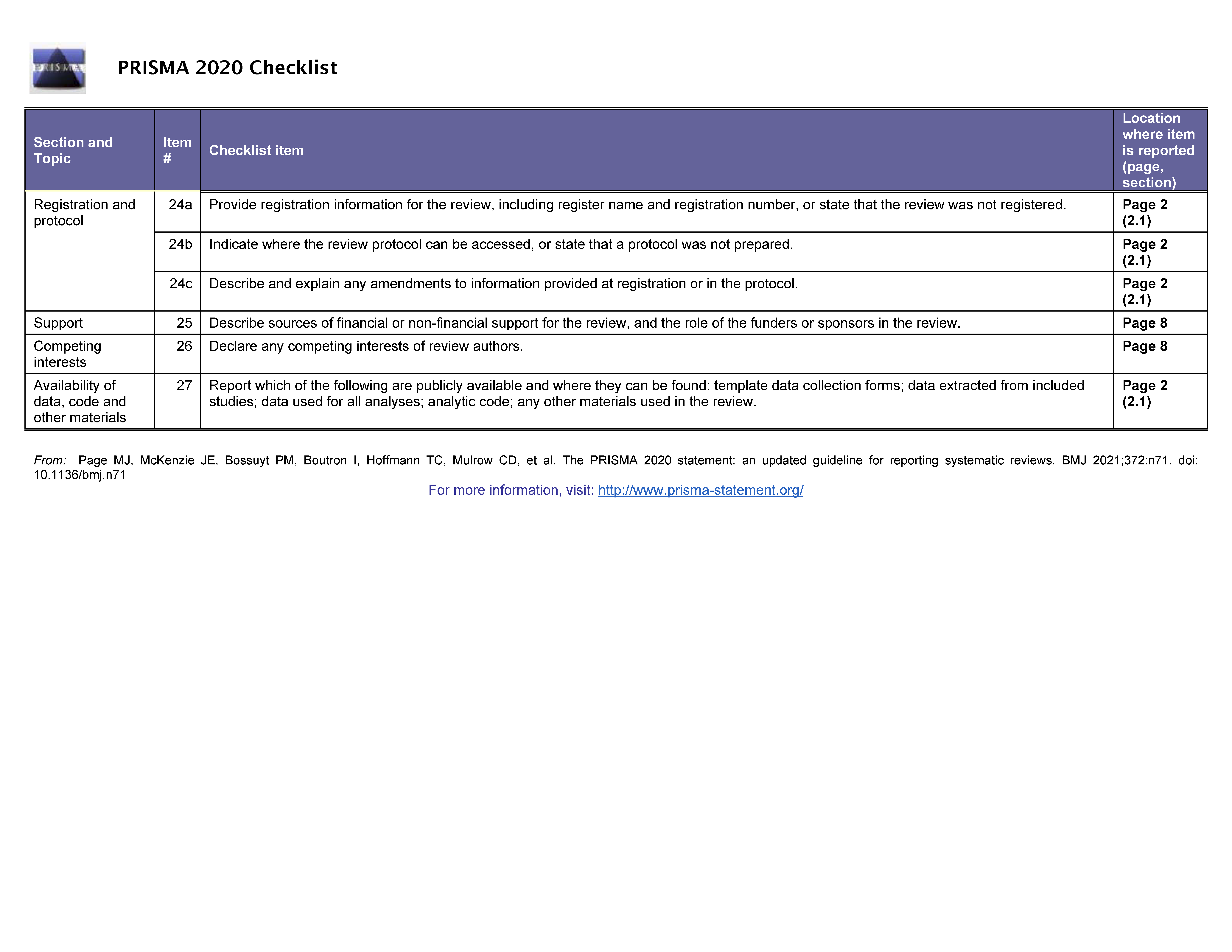

Supplement: Supplementary file 1 [file 2153-8174-23-6-208-s1.zip › Supplementary Figure 3 - PRISMA CHECLIST.tiff]

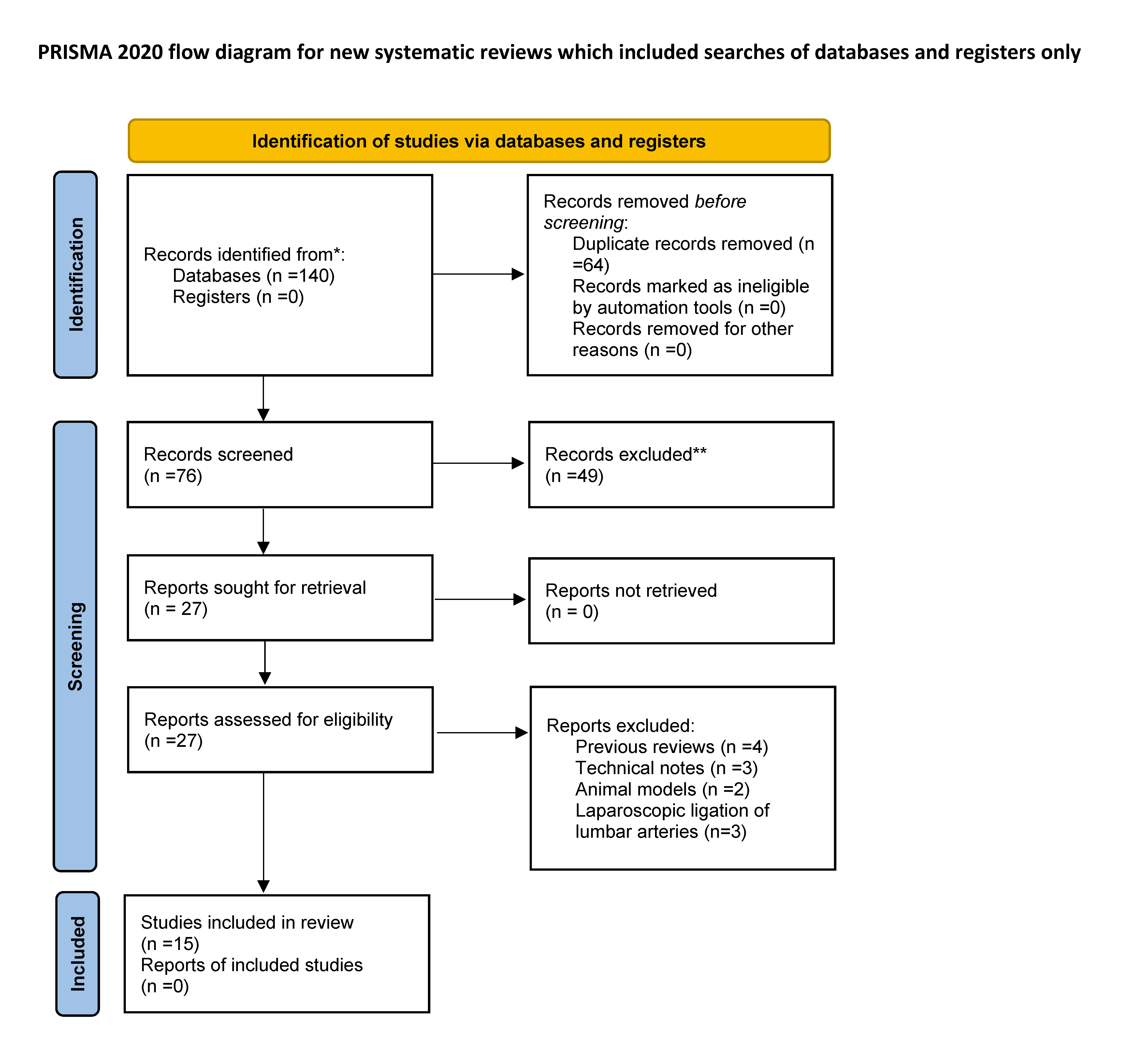

Supplement: Supplementary file 1 [file 2153-8174-23-6-208-s1.zip › Supplementary Figure 4 - PRISMA Flow chart.tif]
